# The natural history of transfer RNA and its interactions with the ribosome

**DOI:** 10.3389/fgene.2014.00127

**Published:** 2014-05-09

**Authors:** Gustavo Caetano-Anollés, Feng-Jie Sun

**Affiliations:** ^1^Evolutionary Bioinformatics Laboratory, Department of Crop Sciences, University of IllinoisUrbana-Champaign, IL, USA; ^2^School of Science and Technology, Georgia Gwinnett CollegeLawrenceville, GA, USA

**Keywords:** structure, phylogenetic analysis, sequence, non-coding RNA, translation, ribosome, origin of life

Transfer RNA (tRNA) is undoubtedly the most central and one of the oldest molecules of the cell. Without it genetics and coded protein synthesis are impossible. The crucial specificities responsible for the genetic code and accurate translation are by far entrusted to interactions between tRNA and translation proteins, fundamentally aminoacyl-tRNA synthetase (aaRS) enzymes and elongation factor (EF) switches (Yadavalli and Ibba, 2012). Discrimination mediated by aaRSs and EFs against misincorporated tRNA and amino acids is at least 20 times more stringent than ribosomal recognition, editing, and other proofreading mechanisms (Reynolds et al., 2010). The fact that crucial genetic code specificities in highly selective interactions with protein enzymes do not involve the ribosomal ribonucleoprotein biosynthetic machinery challenges the “replicators first” origin of life scenario of an ancient RNA world (Caetano-Anollés and Seufferheld, 2013). It also highlights the central functional, mechanistic, and evolutionary roles of tRNA and its recognition determinants, which enable coevolution between nucleic acids and proteins. These coevolutionary relationships are compatible with a late origin of the ribosome in its mechanism and not in protein biosynthesis, which was inferred from the computational analysis of thousands of RNAs and proteomes (Harish and Caetano-Anollés, 2012). These analyses showed tight coevolution of ribosomal RNA (rRNA) and ribosomal proteins (r-proteins). While these relationships delimit molecular makeup when organisms use translation to negotiate growth and viability amidst environmental change, coevolution also constrains recruitment of the canonical L-shaped structure of the tRNA molecule into a multiplicity of modern functions. These new functions include the synthesis of antibiotics, bacterial cell wall peptidoglycans and tetrapyrroles, modification of bacterial membrane lipids, protein turnover, and the synthesis of other aminoacyl-tRNA molecules (Francklyn and Minajigi, 2010). Here we unfold coevolutionary relationships between tRNA substructures and translation proteins that embody crucial protein-nucleic acid interactions. We focus on a series of computational biology analyses of the structure and conformational diversity of tRNAs and their interacting proteins that provide information about the history of structural accretion of this “adaptor” molecule. Using this information, we place tRNA history within the framework of an evolutionary timeline of protein domain innovation, uncovering the natural history of tRNA within the context of the geological record.

## tRNA molecules are old and evolve by accretion of structural parts

**Figure 1:**
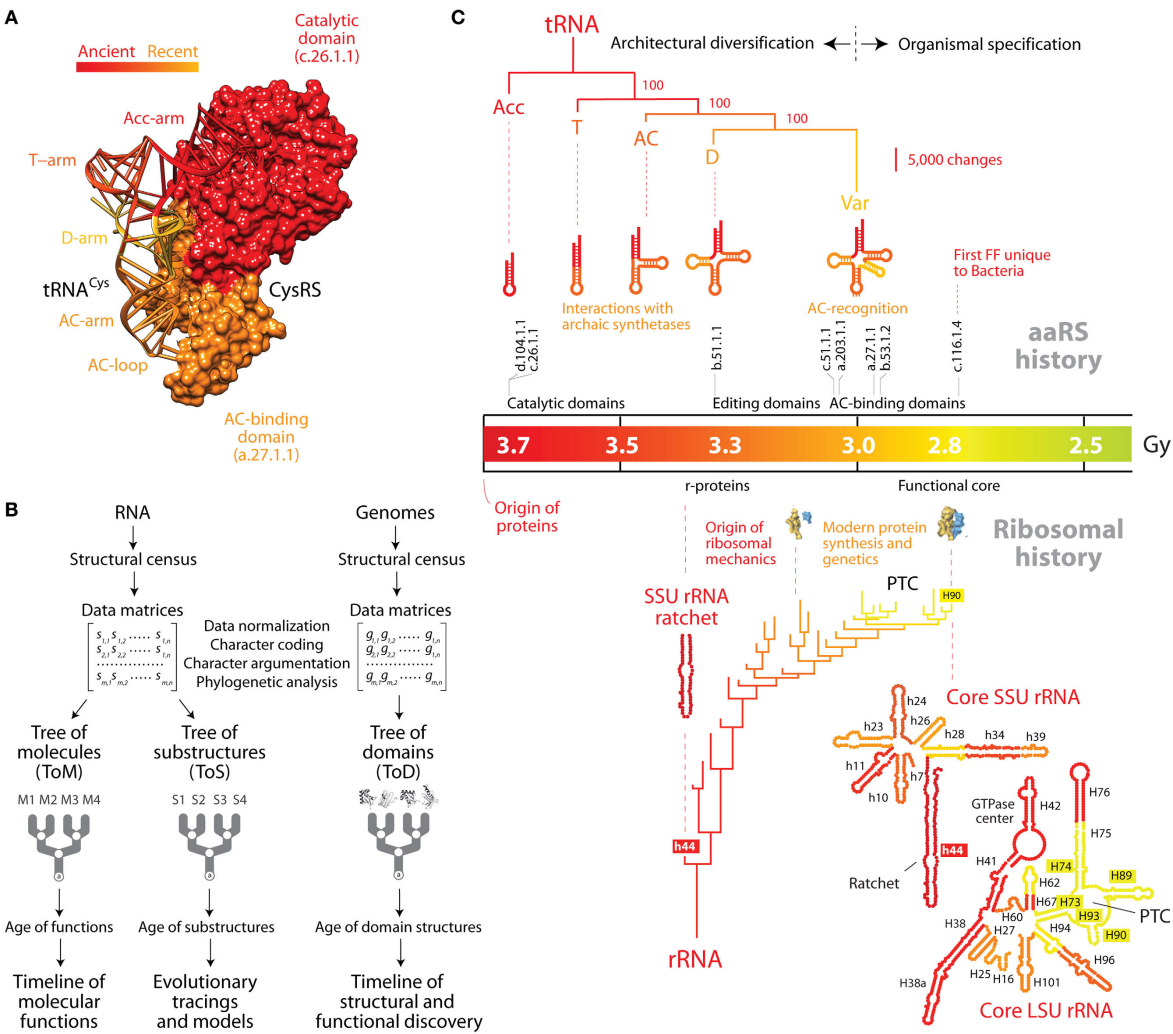
**The natural history of tRNA inferred from nucleic acid-protein interactions and structural phylogenomics. (A)** The history of tRNA portrays the history of its interactions with cognate aminoacyl-tRNA synthetase (aaRS) protein enzymes. This is exemplified by the domains of the tRNA and cysteinyl-tRNA synthetase binary complex (PDB entry 1U0B), which are colored according to their age. The ancient “top half” of tRNA embeds a “operational code” in the identity elements of the acceptor arm that interact with the catalytic domain of aaRSs through classes I and II modes of tRNA recognition. The evolutionarily recent “bottom half” of tRNA holds the standard code in identity elements of the anticodon loop that interact with anticodon-binding domains of aaRSs. **(B)** Flow diagram showing the retrodiction strategy used to build phylogenetic trees of RNA molecules (ToMs) and associated trees of substructures (ToSs), and trees of protein domains (ToDs). The structures of RNA molecules are first decomposed into substructures. Structural features of substructures such as helical stem tracts and unpaired regions are coded as phylogenetic characters and assigned character states according to an evolutionary model that polarizes character transformation toward an increase in conformational order (character argumentation). Coded characters (s) are arranged in data matrices, which can be transposed. Phylogenetic analysis using maximum parsimony optimality criteria generates rooted ToMs and ToSs. A census of domain structures in proteomes of hundreds of completely sequenced organisms is used to compose data matrices, which are then used to build ToDs. Elements of the matrix (g) represent genomic abundances of domain structures in proteomes, defined at different levels of classification of domain structure (e.g., SCOP folds, superfamilies, and families). They are converted into multi-state phylogenetic characters with character states transforming according to linearly ordered and reversible pathways. Embedded in the trees of nucleic acids and proteins are timelines that assign age to molecular structures and associated functions. **(C)** The natural history of tRNA and rRNA overlap when they are mapped onto a timeline of protein domain history. A tree of tRNA substructures (ToS) was derived from statistical phylogenetic characters that define a molecular morphospace (the Shannon entropy of the base-pairing probability matrix, base-pairing propensity and mean length of stem structures) in 571 tRNA molecules. The optimal most parsimonious tree (43,281 steps; consistency index = 0.853, retention index = 0.654, rescaled consistency index = 0.557, g_1_ = −1.033) was recovered from a branch-and-bound search. The most basal subtree of a ToS describing the evolution of the rRNA core (Harish and Caetano-Anollés, 2012) is also shown. Both trees are anchored to the geological record via an evolutionary timeline of first appearance of protein domains that are capable of establishing crucial interactions with the RNA molecules (see description in the main text). AC, anticodon; PTC, peptidyl transferase center.

When studying the organismal distribution of a catalog of over a thousand RNA families describing the modern RNA world, tRNA was found to be one of only five families that were universally present (Hoeppner et al., 2012). These families showed a strong vertical evolutionary trace and included rRNA and ribonuclease P (RNase P) RNA, which are present (with exceptions; e.g., Randau et al., 2008) in all studied cellular organisms and are minimally affected by horizontal gene transfer. We note however that RNA-free RNase P (Gutmann et al., 2012; Taschner et al., 2012) can challenge RNase P RNA ancestrality (Sun and Caetano-Anollés, 2010). The ubiquity of tRNA in the cellular lineages of life and its central molecular role provide strong support to the very early origin of the molecule, prompting the study of the origin and evolution of the tRNA molecule using information in its sequence and structure (Fitch and Upper, 1987; Eigen et al., 1989; Di Giulio, 1994; Sun and Caetano-Anollés, 2008a; Farias, 2013). A computational analysis of the history of tRNA based on the structure of thousands of molecules revealed that tRNAs evolve by accretion of component parts (substructures) and that the “top half” of tRNA that includes the acceptor stem is more ancient that the “bottom half” with its anticodon arm (Sun and Caetano-Anollés, 2008a; reviewed in Sun and Caetano-Anollés, 2008b) (Figure 1A). While other models of evolutionary growth of the tRNA molecule have been proposed (Di Giulio, 2012), phylogenetic reconstructions are compatible with biochemical evidence of molecular recognition that makes amino acid charging ancestral and molecularly distant (~70 Å) to codon recognition, which locate to more modern regions of tRNA (Caetano-Anollés et al., 2013). These findings revive the “genomic tag” hypothesis in which tRNA harbored ancestral genomic information and the derived bottom half provided genetic code specificity (Weiner and Maizels, 1987).

## Phylogenomic retrodiction uncovers coevolution between tRNA substructures and interacting aaRS protein domains

In the studies mentioned above, phylogenetic analysis of nucleic acid structure was directly derived from structural topology and the thermodynamics of tRNA (Caetano-Anollés, 2002a,b; Sun et al., 2007; Sun and Caetano-Anollés, 2008a), taking unique advantage of links that exist between secondary structure and conformation, dynamics, and adaptation (Bailor et al., 2010). Specifically, a census of geometrical features that describe the length and topology of tRNA substructures (such as stem and non-paired segments) or statistical features describing their stability and conformational diversity were analyzed with modern phylogenetic methods to produce phylogenetic trees of molecules (ToMs) and trees of substructures (ToSs) that portray the history of the system (molecules) or its component parts (substructures), respectively. Figure 1C shows a ToS that describes the evolution of stem substructures of the tRNA molecule and of early evolving stem substructures of rRNA. The trees that are produced are rooted using a phylogenetic process model that complies with Weston's generality criterion. The model automatically roots the trees by assuming conformational stability increases in evolution as structures become canalized (Sun et al., 2010). The validity of polarization and rooting depends on the axiomatic component of character transformation, which is falsifiable and supported by considerable evidence (e.g., thermodynamic and phylogenetic; Sun et al., 2010).

While ToSs are powerful retrodiction statements that unfold history of RNA accretion (Sun and Caetano-Anollés, 2008a,b,c, 2009, 2010; Sun et al., 2007; Harish and Caetano-Anollés, 2012), the gradual appearance of protein domains in evolutionary history can be inferred from phylogenomic trees of domains (ToDs) (Figure 1B) (Caetano-Anollés and Caetano-Anollés, 2003) and can illustrate the establishment of intermolecular interactions in evolution. Domains are structural and evolutionary units of proteins that are highly conserved (Caetano-Anollés et al., 2009). The evolutionary accumulation of these units unfolds recurrence patterns that encompass the entire history of proteins and can be mined with suitable phylogenomic methods. ToDs are derived from a structural census of protein domains in the proteomes of hundreds to thousands of genomes that have been completely sequenced. The fold structures of domains are defined using the different levels of structural abstraction of the accepted classification gold standards, the SCOP (Murzin et al., 1995) or CATH (Orengo et al., 1997) databases. Timelines of domain innovation are then derived directly from the trees taking advantage of their highly imbalanced nature. Imbalance unfolds when the splitting of lineages depends on an evolving “heritable” trait (Heard, 1996). In our case, the evolving trait is the gradual accumulation of domains in proteomes and the semipunctuated discovery of new fold structures (made evident for example in simulations; Zeldovich et al., 2007). The predictive power of ToDs is considerable (Caetano-Anollés and Seufferheld, 2013) and central for the history of tRNA, as ToDs have established the evolutionary history of aaRS domain structures and their associated coevolving tRNA molecules (Caetano-Anollés et al., 2013). The timeline of evolutionary appearance of fold families revealed the early emergence of the “operational” RNA code linked to the specificities of synthetases that were homologous to the catalytic domains of modern TyrRS and SerRS protein enzymes. These archaic synthetases interacted with the “top half” of tRNA and were capable of peptide bond formation and aminoacylation (Caetano-Anollés et al., 2013). The timeline also showed the late implementation of the standard genetic code with the late appearance of anticodon-binding domains that interacted with the “bottom half” of tRNA. Figure 1A shows a representative aaRS enzyme and the tight coevolutionary link between aaRS domains and tRNA arms. Remarkably, structural phylogenomic retrodictions indicate that genetics arose through episodes of structural recruitment as an exacting mechanism that favored flexibility and folding of the emergent proteins (Caetano-Anollés et al., 2013). These enhancements of phenotypic robustness matched evolutionary trends of folding speed in proteins (Debes et al., 2013) and are compatible with recent simulations of the origin of the genetic code (Jee et al., 2013).

## Abundance of protein domains in proteomes follows an evolutionary clock

The history of RNA does not represent a phylogenetic statement that applies to the entire world of RNA molecules. Consequently, it cannot be placed within a global historical context. In contrast, the history of protein domains inferred from ToDs follows a global molecular clock of fold structures that spans 3.8 billion years (Gy) of evolution (Wang et al., 2011). Traditionally, molecular clocks are based on rates of change in protein or nucleic acid sequences, which are limited by historical information existing in the individual protein or nucleic acid molecules being studied (Zuckerkandl and Pauling, 1965; Ayala et al., 1998). These clocks are therefore constrained by the highly dynamic nature of sequence change, including the problems of mutational saturation and rate heterogeneity (heterotachy). In contrast, molecular structures exhibit characteristics of recurrent change that are much more stable. The clocks of domain structures were calibrated by associating diagnostic domain structures with multiple geological ages derived from the study of fossils and microfossils, geochemical, biochemical, and biomarker data. Remarkably, excellent linear correlations between the ages of domain structures at fold and fold superfamily levels of SCOP and geological timescales were identified and used to time fundamental evolutionary events (Wang et al., 2011). These events included the rise of planetary oxygen and episodes of organismal diversification (Wang et al., 2011; Kim et al., 2012).

## The cloverleaf structure of tRNA unfolds early in evolution, prior to the appearance of a functional ribosomal machinery

Assuming that the age of interactions that are established between RNA and proteins is the age of the interacting components, we tracked the appearance of domains in ribonucleoprotein complexes along the evolutionary timeline and used the molecular clock of folds to link interactions to a geological timescale (Figure 1C). The catalytic domains of classes I and II aaRS enzymes (belonging to SCOP families d.104.1.1 and c.26.1.1, respectively) are the first to appear in the timeline ~3.7 Gy ago (Caetano-Anollés et al., 2013). These domains harbor pre-transfer and post-transfer editing and trans-editing activities. The most ancient of these editing structures, present in the catalytic domains of TyrRS, SerRS, and LeuRS, involve interactions with the oldest type II cognate tRNAs, which harbor a long variable loop necessary for tRNA recognition (Sun and Caetano-Anollés, 2008c). While the evolutionary significance of the variable loop in tRNA-aaRS interactions is unclear (Sun and Caetano-Anollés, 2008c), its late evolutionary appearance could simply represent the shift or recruitment of an archaic interacting region of the molecule. Interactions of tRNA with the “ValRS/IleRS/LeuRS editing” domain (SCOP family b.51.1.1) (Hale et al., 1997) suggest the D arm was already present ~3.3 Gy ago, which is derived compared to the acceptor stem (Sun and Caetano-Anollés, 2008a). The late appearance of anticodon-binding domains (beginning with SCOP family c.51.1.1) in well over half of aaRSs ~3 Gy ago confirms that the full “bottom half” of tRNA and its anticodon loop identity elements unfolded completely before the onset of planetary oxygenation and cellular diversification ~2.9 Gy ago.

Comparing the natural history of tRNA (Sun and Caetano-Anollés, 2008a) and the ribosome (Harish and Caetano-Anollés, 2012) within the framework of the interacting proteins shows the remarkable functional connection of the cloverleaf structure and ribosomal functionality (Figure 1C). The origin of r-proteins in interaction with helix 44 (the ribosomal ratchet) of the small subunit (SSU) rRNA occurred 3.3–3.4 Gy ago once the tRNA molecule unfolded its anticodon arm. This manifests in the pivotal role of one of the two earliest r-proteins, S12, in tRNA selection (anticipated by Ogle and Ramakrishnan, 2005), which is mediated by a bonding network connecting two sites in S12 to the anticodon and the CCA arm of the tRNA-elongation factor bound state (Li et al., 2008). Similarly, the full cloverleaf structure of tRNA was already present when the ribosomal peptidyl transferase center (PTC) responsible for modern protein synthesis appeared in the emerging domain V of the large subunit of rRNA 2.8–3.1 Gy ago. This is an expected outcome since the structurally mature 70–80 Å-long and 20–25 Å-wide tRNA molecule must traverse a path of ~100 Å and physically span the intersubunit interface of the ribosomal core for the ensemble to be fully functional (Agirrezabala and Frank, 2009). Remarkably, this late development of the ribosomal core coincided with the appearance of pathways of amino acid (Kim et al., 2012) and purine nucleotide biosynthesis (Caetano-Anollés and Caetano-Anollés, 2013). This suggests that tRNA and ribosomal functionality (anticodon loop recognition, decoding, protein biosynthesis) and modern metabolic pathways for amino acids and nucleotides developed concurrently, supporting the co-evolution theory of the genetic code (Wong, 2005).

## Conclusion

The natural and overlapping history of tRNA and rRNA reveals that: (1) the tRNA cloverleaf structure unfolded prior to the appearance of a fully functional ribosomal core, (2) the primordial role of tRNA, originally linked to archaic dipeptide-forming synthetases, was coopted into modern translation functions once anticodon-loop specificities appeared concurrently with the PTC, and (3) the emergence of modern genetics unfolded relatively quickly in a period of 0.3–0.5 Gy, starting with anticodon-loop recognition and once the cloverleaf structure had formed.

### Conflict of interest statement

The authors declare that the research was conducted in the absence of any commercial or financial relationships that could be construed as a potential conflict of interest.
